# Platelet-to-Lymphocyte Ratio—A Real or Fake Bridge Between Inflammation and Coagulation in COVID-19 Patients: A Scoping Review

**DOI:** 10.3390/diagnostics16101476

**Published:** 2026-05-13

**Authors:** Maja Aleksandra Oksentowicz, Maria Sztachelska, Violetta Dymicka-Piekarska

**Affiliations:** 1 Department of Clinical Laboratory Diagnostics, Medical University of Bialystok, 15-089 Bialystok, Poland; 41680@student.umb.edu.pl; 2 Centre of Regenerative Medicine, Medical University of Bialystok, 15-089 Bialystok, Poland; maria.sztachelska@umb.edu.pl

**Keywords:** COVID-19, platelet-to-lymphocyte ratio, COVID-19-associated coagulopathy, receiver operating characteristic, scoping review

## Abstract

**Background:** Patients with COVID-19 often develop COVID-19-Associated Coagulopathy (CAC)—an imbalance between procoagulant and anticoagulant pathways resulting from the uncontrolled inflammatory response triggered by SARS-CoV-2 infection. This study aims to investigate the impact of a hematological and inflammatory parameter—the platelet-to-lymphocyte ratio (PLR)—on the severity and mortality of COVID-19. **Methods:** We conducted a comprehensive search of the PubMed database and yielded 75 articles published in the period of 2020–2025, of which 20 studies that evaluated the prognostic value of PLR on hospital admission in COVID-19 patients were included. The review particularly focuses on ROC analyses and reported AUC values. **Results:** A total of 20 studies were analyzed, including 13 studies assessing disease severity and 14 studies evaluating mortality. Higher PLR values have been observed in patients with a more severe course of COVID-19 compared to those with milder disease, and in non-survivors compared to survivors. However, the literature shows inconsistency regarding the diagnostic utility of PLR based on ROC curve analysis. The reported AUC values ranged from 0.559 to 0.811 for disease severity differentiation and from 0.474 to 0.758 for mortality, which may be related to the heterogeneity of the study populations included in the analysis. **Conclusions:** PLR may not serve as a direct bridge between inflammation and coagulation in COVID-19-Associated Coagulopathy, but it is indirectly linked to disease severity and mortality, as it reflects changes in both platelet and lymphocyte counts. It is a complementary marker that may assist clinicians in assessing COVID-19 patients but still requires further investigation.

## 1. Introduction

An inevitable consequence of human development is the emergence and evolution of infectious agents whose pathogenicity and virulence can surpass the body’s natural defense mechanisms. This poses a significant global risk for the spread of diseases, potentially leading to epidemics and pandemics. There has been an alarming number of Severe Acute Respiratory Syndrome Coronavirus 2 (SARS-CoV-2) cases and deaths worldwide since the first case was identified in December 2019 in Wuhan, Central China. The outbreak was officially declared a pandemic by the World Health Organization (WHO) on 11 March 2020 [1,2,3,4]. To date, nearly 800 million confirmed cases of COVID-19 have been reported globally, including over 7 million deaths [5,6].

Viral infections are often associated with changes in the coagulation system. COVID-19 patients develop immuno-thrombosis, i.e., a state of mutual reinforcement of inflammation and coagulopathy, which is referred to as COVID-19-Associated Coagulopathy (CAC). Therefore, it is essential to closely examine the underlying mechanisms of disease progression and its complications, as well as to identify biomarkers that could facilitate diagnosis and help assess their dynamics. To thoroughly investigate the mechanisms of hemostasis disorders, it is important to note that these complications are rooted in an imbalance between procoagulant and anticoagulant processes, as well as in the interplay between inflammation and coagulation. Therefore, particular attention should be paid to changes in platelets and leukocytes, especially lymphocyte parameters [7,8,9,10,11].

### 1.1. COVID-19-Associated Coagulopathy (CAC)

Under pathological conditions associated with systemic inflammation caused by SARS-CoV-2 infection, various immune cells become involved, primarily leukocytes and platelets. Uncontrolled and excessive pro-inflammatory activity leads to endothelial damage, causing endothelial cells to express higher levels of procoagulant factors such as collagen, fibronectin, and tissue factor (TF). This promotes platelet adhesion to the vascular endothelium, while the physiological mechanisms that normally inhibit platelet adhesion and aggregation—such as prostacyclin, nitric oxide, tissue factor pathway inhibitor (TFPI), and thrombomodulin—fail to counterbalance the procoagulant factors. Activated platelets aggregate through fibrinogen bridges formed via GP IIb/IIIa and release granule contents, including thrombin, Ca^2+^, von Willebrand factor (vWF), thromboxane A_2_ (TXA_2_), and platelet factor 4 (PF4), which are mediators that enhance platelet and leukocyte chemotaxis and platelet–immune cell interactions [12,13]. Elevated expression of P-selectin enables platelets to bind PSGL-1 on endothelial cells after vascular injury and on leukocytes, leading to the formation of leukocyte–platelet aggregates (LPAs) [14]. Activated platelets also display CD40L, which supports leukocyte recruitment and extravasation, as well as C-type lectin-like receptor 2 (CLEC-2), whose ligand is present on cells such as macrophages [15,16]. In addition, thrombin generation and fibrin formation strengthen leukocyte–platelet interactions and promote mutual activation [13].

This cascade leads to intravascular coagulation and formation of aggregates known as immuno-thrombi, composed mainly of platelets and leukocyte, which increase the risk of turbulent blood flow and elevated shear stress [17,18]. This results in dysregulated activation of the coagulation cascade, leading to the development of thrombo-inflammation, referred to as COVID-19-Associated Coagulopathy [11]. CAC is a condition characterized by a vicious cycle of inflammation and coagulopathy caused by dysregulation of multiple pathways, predisposing individuals infected with SARS-CoV-2 to a higher risk of thromboembolic complications—both venous and arterial—and thrombotic microangiopathy, particularly in the pulmonary vasculature [19,20]. Patients with severe SARS-CoV-2 infection may develop lower extremity deep vein thrombosis (DVT), acute cerebrovascular disease (ACD), ischemic strokes, acute coronary syndrome (ACS), myocardial infarction, and other broadly defined thromboembolic events, as well as systemic complications such as multiple organ dysfunction syndrome (MODS) or disseminated intravascular coagulation (DIC). It is a multifactorial process linked to more adverse clinical outcomes in patients hospitalized with COVID-19 [7,21]. Each of these conditions ultimately contributes to a poor prognosis and an increased risk of death [9]. The uncontrolled amplification of inflammation and coagulation is closely linked to increased disease severity and mortality in COVID-19 patients [22].

Changes in coagulation and inflammatory responses induced by SARS-CoV-2 infection are reflected in alterations in platelet and lymphocyte counts. As PLR captures both platelet activation and lymphocyte dynamics, it may serve as an indirect indicator of these processes. Therefore, particular attention should be given to changes in these components as the key constituents of PLR in the context of COVID-19-Associated Coagulopathy.

### 1.2. Platelets and Lymphocytes During COVID-19

COVID-19 leads to changes in numerous parameters, including biochemical and hematological ones, such as platelet indices, leukocyte counts, and their subpopulations.

Inflammation primarily affects platelet count (PLT), size, and maturity [23]. According to the current literature, changes in thrombocyte parameters represent a significant, non-specific prognostic marker during COVID-19 infection. Damage to the pulmonary capillary beds caused by SARS-CoV-2 is accompanied by a decrease in platelet count, which may result from the involvement of platelets in combating the infection and disturbances in hemostasis [24]. A hallmark of COVID-19 is the cytokine storm, characterized by excessive release of pro-inflammatory cytokines (IL-6, IL-8, IL-1β, and TNF-α), which can cause platelet hyperactivation. It may also result from PAMPs and DAMPs interacting with PRRs, antibodies produced during infection, and direct viral interaction with platelet receptors such as ACE2 or CD147. Additionally, the virus can infect bone marrow megakaryocytes, and viral particles have been detected in circulating platelets. In response to increased platelet consumption due to lung injury and increased coagulation activity, thrombocytopenia is commonly observed in patients with COVID-19 [21,25,26,27,28].

During COVID-19, as in other viral infections, adaptive immunity involving T and B lymphocytes plays a key role in combating the virus [29]. Antigen-presenting cells, such as dendritic cells, process and present specific viral proteins. These cells convey information to naive T lymphocytes, which become activated, mature, and differentiate in lymphoid nodes into CD^4+^ and CD^8+^ T cells. Th lymphocytes (CD^4+^) stimulate B lymphocytes to proliferate and differentiate into plasmacytes that produce antibodies against viral antigens. Additionally, Th lymphocytes secrete cytokines such as IL-2, IL-4, and IL-5 and antiviral IFN-γ, which further support the immune response [30]. Tc lymphocytes secrete cytotoxic proteins in response to detection of viral antigens on the surface of infected cells via MHC class I receptors mainly in the pulmonary capillary beds [31]. During SARS-CoV-2 infection, lymphopenia frequently occurs, characterized by a significant decrease in CD^8+^ T cells, and is associated with severe COVID-19 [32]. This is also primarily associated with the consumption of immune cells during the systemic inflammatory response triggered by the virus. Additionally, there is significant migration of cytotoxic T lymphocytes (Tc) mainly to the lung tissue, where the damage and destruction of cells caused by SARS-CoV-2 lead to the release of large amounts of cytokines, PAMPs, DAMPs and reactive oxygen species (ROS) [33]. A key factor contributing to the overall lymphocyte depletion may also be the characteristic cytokine storm. High levels of pro-inflammatory cytokines, including IL-6 and TNF-α, have been linked to lymphopenia and can directly contribute to lymphocyte depletion. Additionally, viruses themselves can directly induce lymphocyte apoptosis, disrupt lymphopoiesis in the bone marrow and directly destroy lymphatic organs [34].

Thrombocytopenia and lymphopenia are among the most common laboratory findings in critically ill COVID-19 patients [35]. Total platelet and lymphocyte counts were lower in severe or critical illness than in mild or moderate courses of COVID-19. Khalid et al. [36] conducted a study involving 487 patients with confirmed COVID-19 and 300 healthy controls, demonstrating that platelet and lymphocyte counts differed significantly between the two groups. The comparison of these parameters among subgroups with varying disease severity was also statistically significant. Furthermore, two meta-analyses—one focusing on platelet count and the other on lymphocyte count—demonstrated that thrombocytopenia and lymphopenia on admission were both associated with poor outcomes in patients with COVID-19 [32,37].

An indicator reflecting both increased platelet activation and consumption in coagulation processes, as well as lymphocyte involvement in the ongoing inflammatory response, is the non-specific inflammatory marker known as the platelet-to-lymphocyte ratio (PLR). During SARS-CoV-2 infection, platelet and lymphocyte counts decrease considerably due to both the direct impact of the virus and systemic changes occurring in the body, which affects the PLR. Elevated PLR values have already been observed in patients with chronic inflammatory conditions such as cancers, cardiovascular diseases, viral infections, autoimmune diseases and other pulmonary diseases as a pulmonary embolism [38,39,40,41,42,43]. The PLR allows for the simultaneous assessment of both the humoral immune response and hemostatic function, which is particularly relevant in the context of COVID-19-Associated Coagulopathy. Since its components include both platelets, reflecting the coagulation process, and lymphocytes, reflecting the inflammatory response, PLR may be considered an indirect link between inflammation and coagulation in COVID-19-Associated Coagulopathy [10].

In this scoping review, we analyzed studies that reported PLR values at the time of hospital admission and evaluated the association between the PLR and clinical outcomes in COVID-19. We focused on the potential of the PLR to predict disease severity and mortality during the SARS-CoV-2 infection. We primarily focused on receiver operating characteristic (ROC) analysis and reported area under the curve (AUC) values in relation to COVID-19 severity or mortality, as this is a fundamental method for assessing diagnostic performance and is widely used in the evaluation of biomarkers.

## 2. Materials and Methods

### 2.1. Study Design

To explore the relationship between the platelet-to-lymphocyte ratio (PLR) and the severity and the mortality of COVID-19, we conducted a scoping review following Arksey and O’Malley’s methodological framework for scoping reviews [44]. This framework comprises five key steps that guided our analysis: identifying the research question, identifying relevant studies in the literature, selecting relevant studies, charting the data, collating and summarizing, and reporting the findings.

### 2.2. Search Strategy

A systematic search was conducted in October 2025 using the PubMed database (National Library of Medicine, Bethesda, MD, USA; https://pubmed.ncbi.nlm.nih.gov/) with keywords including platelet-to-lymphocyte ratio, COVID-19, SARS-CoV-2, severity and mortality (Table 1).

The primary objective of this scoping review was to evaluate the diagnostic value of the platelet-to-lymphocyte ratio (PLR) in COVID-19 patients and its utility in predicting disease severity and mortality.

### 2.3. Inclusion Criteria

We included studies involving patients with confirmed COVID-19 from various countries, specifically those that examined the PLR on hospital admission and assessed its association with disease severity and mortality. The inclusion criteria for the scoping review were as follows: studies that compared PLR values measured at hospital admission (based on the first routine blood examination) with subsequent COVID-19 severity and mortality outcomes, including the use of receiver operating characteristic (ROC) analysis of the PLR to discriminate between different levels of disease severity and mortality risk. Only articles published in English between 2020 and 2025, involving adult human subjects, and accessible in full text were considered.

### 2.4. Exclusion Criteria

Exclusion criteria comprised: non-research articles such as protocols, books, reviews, meta-analyses, letters to the editor, and studies that stratified patients based solely on comorbidities without direct PLR analysis related to COVID-19 severity or mortality.

### 2.5. Quality Assessment

Two independent reviewers screened the records by assessing titles and abstracts to identify relevant studies. In cases of disagreement, a third reviewer was consulted to reach consensus.

### 2.6. Statistical Analysis

**Figure 1:**
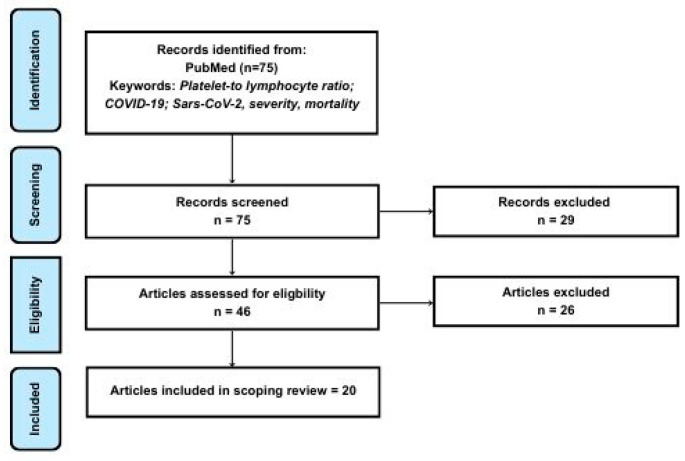
Literature search flow diagram (based on the Preferred Reporting Items for Systematic Reviews and Meta-Analyses: Extension for Scoping Reviews (PRISMA-ScR)).

The scoping review was conducted in accordance with the Preferred Reporting Items for Systematic Reviews and Meta-Analyses: Extension for Scoping Reviews (PRISMA-ScR) guidelines (Figure 1) [45]. Data extraction was subsequently performed using a standardized Excel form, capturing information including authorship, country, patient numbers in both control and study groups, PLR values at hospital admission, and outcomes related to disease severity and mortality. The results of searching were exported into EndNote, where duplicate records were removed. A formal assessment of the methodological quality and risk of bias of the included studies was not performed.

### 2.7. Charting the Data

The initial literature search yielded 75 articles from the database. The first step involved a primary screening of titles and abstracts to select studies that focused on the relationship between inflammatory markers and the severity and/or mortality of COVID-19 patients. To enhance consistency, all three authors collaborated to identify the articles most relevant to our research question. Severe cases were defined as patients presenting with clinical symptoms such as dyspnea and respiratory distress, requiring mechanical ventilation, experiencing combined organ failure, or requiring admission to the intensive care unit (ICU). For the severity group, we included studies that used PLR as a predictor of intubation, ICU admission, or septic shock. Subsequently, full-text screening was conducted to assess study eligibility. Priority was given to studies that performed ROC analysis and reported AUC values in relation to COVID-19 severity or mortality. We also included the ROC to define the optimal cut-off, sensitivity, and specificity of PLR to determine COVID-19 severity and mortality. We also included the ROC to define the optimal cut-off, sensitivity, and specificity of PLR to determine COVID-19 severity and mortality.

In total we received 20 articles. Thirteen articles reported PLR in severe and non-severe COVID-19 patients at hospital admission, while 14 articles analyzed PLR at admission in relation to survival status, comparing survivors and deceased individuals.

## 3. Results

The initial literature search identified 75 articles in the PubMed database using predefined keywords. All records were screened to exclude studies that did not investigate changes in PLR in relation to COVID-19 severity and mortality. During the eligibility assessment, 46 full-text articles were evaluated; however, 26 were excluded due to the absence of ROC curve analysis and AUC values (Figure 1).

Studies were excluded if they focused on highly specific patient subgroups, including those with particular comorbidities, as such populations were not representative of the general COVID-19 cohort considered in this review. We also excluded studies evaluating the diagnostic value of the PLR during different waves of the COVID-19 pandemic, as temporal variations in disease characteristics and management could affect comparability. Furthermore, studies restricted to specific populations, such as elderly individuals or pregnant women, were not included to maintain greater consistency across the analyzed cohorts. These criteria were applied to ensure a more homogeneous study population and to improve the comparability of AUC values across the included studies.

**Figure 2:**
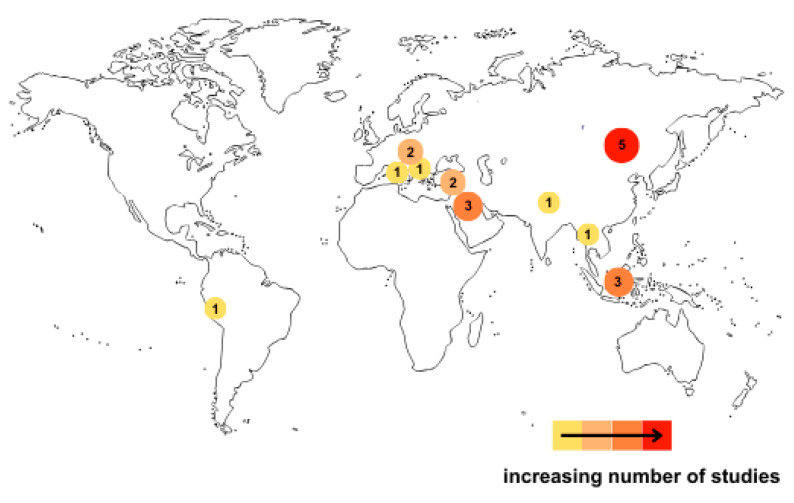
Geographic distribution of the included studies. Note: Color intensity represents the number of studies included from each country. Number of trials: China (*n* = 5), Indonesia (*n* = 3), Iran (*n* = 3), Romania (*n* = 2), Turkey (*n* = 2), India (*n* = 1), Greece (*n* = 1), Peru (*n* = 1), Thailand (*n* = 1), and Italy (*n* = 1).

A total of 20 studies were included—with 13 articles encompassing 4689 patients for disease severity assessment and 14 articles including 5119 patients for mortality assessment—that performed receiver operating characteristic (ROC) analysis and reported area under the curve (AUC) values in relation to COVID-19 severity or mortality. The articles originated primarily from China (*n* = 5), Indonesia (*n* = 3), and Iran (*n* = 3) (Figure 2).

**Figure 3:**
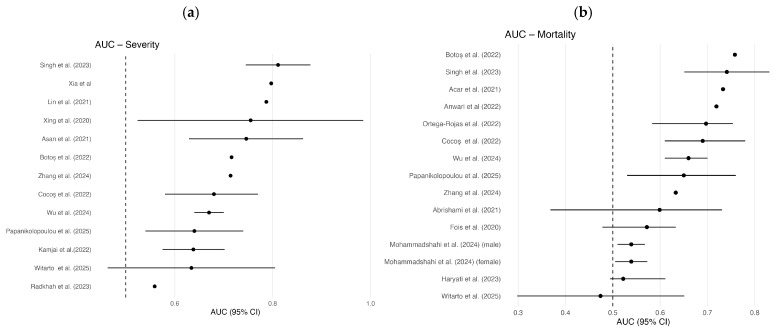
Forest plot presenting the area under the curve (AUC) values from studies evaluating the predictive value of the platelet-to-lymphocyte ratio (PLR) for (**a**) COVID-19 severity and (**b**) COVID-19 mortality. Note: Horizontal lines represent the 95% confidence intervals (95% CI), and dots indicate the point AUC estimates for each study.

Data extracted from the selected studies were summarized in tables including the author, year of publication, country, PLR values in control and study groups, as well as AUC, 95%CI, sensitivity (%), specificity (%), cut-off, and *p*-value (Table 2 and Table 3). For graphical comparison of the AUC values, forest plots were generated separately for studies focusing on disease severity and mortality in COVID-19 patients (Figure 3).

The review indicates that PLR demonstrates variable clinical relevance as an indirect, non-specific biomarker measured at hospital admission for assessing both disease severity and mortality risk in COVID-19, with findings differing across studies, thus highlighting the need for further research. Although some evidence suggests acceptable performance, other studies report limited discriminatory ability, in some cases below the level expected for a clinically useful marker. Overall, the available data indicate that PLR values tend to increase with greater disease severity and higher mortality risk.

### ROC Curve Analysis

Analysis of ROC curves and AUC values (Figure 3) illustrates the distribution of the diagnostic performance of PLR across the included studies. AUC values were interpreted according to commonly used criteria: values of 0.9–1.0 are considered excellent, 0.8–0.9 good, 0.7–0.8 fair, 0.6–0.7 poor, and 0.5–0.6 failed. This approach allows for a standardized assessment of the diagnostic accuracy of the evaluated marker across different COVID-19 patient populations.

Our own literature review identified variations in PLR values associated with both COVID-19 severity and mortality. We evaluated the diagnostic performance of the PLR based on the area under the receiver operating characteristic curve. In assessing the diagnostic value of the PLR for predicting COVID-19 severity, we analyzed 13 studies. The lowest AUC value was reported by Radkhah et al. [52] (AUC = 0.559; *p* = 0.001), who evaluated the effectiveness of the PLR in predicting disease severity based on pulmonary thrombotic embolism, with a low sensitivity of 29.94%, a high specificity of 81.46% and a cut-off of 120.5. In contrast, Singh et al. [53] reported a high AUC of 0.811 (*p* = 0.000) with a sensitivity of 70%, a specificity of 75% and a cut-off of 189.2. Another study also demonstrated good diagnostic accuracy with an AUC of 0.797 (*p* < 0.001), sensitivity of 79.12%, and specificity of 81.82%, using a PLR cut-off >230.44 [56].

Our scoping review identified 14 studies reporting AUC values in assessing the PLR’s diagnostic value for predicting COVID-19 mortality. The lowest AUC (0.474) was reported by Witarto et al. [54], with a sensitivity of 86.7% and a specificity of 29.6% at a cut-off of ≥137.674; however, this result was not statistically significant (*p* = 0.783). Higher AUC values were reported by Botoș et al. [47] (AUC = 0.758, cut-off > 428.49) and Acar et al. [60] (AUC = 0.733, sensitivity 57,8% and specificity 68,9%, with cut-off > 289.90), both statistically significant.

In our analysis, we received cut-off values ranging from 120.5 to 428.49 for differentiating disease severity, and from 34.2 to 428.49 for differentiating mortality. Specificity and sensitivity were higher when assessing COVID-19 severity compared to mortality.

Platelet-to-lymphocyte ratio values are not directly comparable across the included studies due to heterogeneity in study populations, including demographic differences, variability in disease severity definitions, and inconsistencies in statistical reporting formats. Despite the application of strict inclusion criteria, the interpretation of PLR values should be undertaken with caution. Nevertheless, these limitations do not negate the potential diagnostic and prognostic value of the PLR.

## 4. Discussion

The PLR is one of the non-specific biomarkers used to assess the prognosis of SARS-CoV-2 infection. Despite the observed variability in AUC values across the studies included in our analysis, the potential diagnostic and prognostic utility of the PLR should not be dismissed. The heterogeneity in study populations, including differences in age distribution, sex stratification, comorbidities, and race and geographical location, likely contributes to the variability in reported results. These factors may influence inflammatory and hematological responses to SARS-CoV-2 infection, thereby affecting PLR values and their predictive performance.

Lymphocyte counts often decline more rapidly than platelet counts, which can lead to a relative increase in the PLR. However, because both components of the ratio may decrease simultaneously during disease progression, PLR values do not always reach markedly elevated levels despite worsening clinical status. Despite the differences in how COVID-19 severity was defined across the analyzed studies, a consistent observation emerges: the PLR tends to increase as the clinical condition of patients worsens. This trend is particularly important because it suggests that, regardless of the specific criteria used to classify disease severity, the PLR may still reflect the progression of the inflammatory and immunological disturbances associated with COVID-19. This situation differs from indices such as the neutrophil-to-lymphocyte ratio (NLR), which are also related to systemic inflammatory status and disease activity and change significantly with worsening COVID-19 prognosis, where one component typically increases (neutrophils) while the other decreases (lymphocytes). Such opposite trends amplify the ratio and may result in a more pronounced increase in the NLR compared with the PLR. Therefore, while focusing on the biological relevance of the PLR is justified, its clinical utility may be more limited than that of the NLR. Consequently, although the PLR may still reflect underlying immune and hematological disturbances in COVID-19, its dynamics can be more complex and may depend on the relative magnitude of changes in both platelet and lymphocyte counts [66,67].

Patients infected with SARS-CoV-2 typically exhibit higher PLR values compared to healthy individuals, which may be associated with the interactions between leukocytes and platelets during COVID-19-Associated Coagulopathy. In a study by Eslamijouybari et al. [68] involving 527 healthy individuals and 527 COVID-19 patients, the PLR was found to be significantly higher in the COVID-19 group. The median PLR values in the control and COVID-19 groups were 104.16 (86.07–125.75) and 172.88 (121.8–236.78), respectively, but receiver operating characteristic analysis assessing the diagnostic value of the PLR showed that the AUC was very low 0.535 (*p* = 0.000). Similar results were obtained by Peng et al. [69], who reported PLR values of 105.87 ± 30.54 in the control group and 169.51 ± 97.51 in COVID-19 patients, a statistically significant difference. When comparing SARS-CoV-2-positive patients with healthy subjects, the AUC was 0.748 (*p* < 0.0001), with a cut-off value of 144.39. Consistently, the PLR remained significantly higher in COVID-19 patients in the study by Sayit et al. [70] with a slightly lower diagnostic value (AUC = 0.669, *p* = 0.000) and a cut-off of 102.8.

A systematic review by Simadibrata et al. [71] confirmed that elevated PLR levels are associated with more severe COVID-19 outcomes. They summarized seven studies with 998 participants (316 of which had severe COVID-19). Lower PLR values on hospital admission were observed in patients with mild to moderate disease progression (SMD: 0.68; 95% CI: 0.43–0.93; I^2^ = 58%). The PLR has been shown to reflect systemic inflammation and is associated with patient prognosis, suggesting that it may serve as a supportive parameter in predicting patient outcomes. Sarkar et al. [72] also confirmed the prognostic value of the PLR in SARS-CoV-2 infection. Their review included 32 studies, featuring a combined total of 2768 non-infected individuals and 3262 participants with COVID-19. According to this analysis, high PLR values at hospital admission were linked to an increased risk of severe disease (MD  = 86.74; 95% CI: 67.7–105.7; I2  =  95%, *p*  <  0.0001) and mortality (MD = 66.10; 95% CI: 47.75–84.44; *p* < 0.00001). These findings highlight the potential of the PLR as a parameter that may support the assessment of disease severity and mortality risk in COVID-19 patients.

### 4.1. Demographic Heterogeneity Across Study Populations

The large variability in AUC values observed across studies included in our analysis may result from the lack of standardization in the study populations. Ortega-Rojas et al. [65] conducted research on patients over 60 years of age, reporting a higher AUC value (0.697), whereas Witarto et al. [54] and Papanikolopoulou et al. [51] included participants aged over 18 years, in whom lower AUC values were observed (0.474 and 0.65, respectively). It is worth noting that only in the study by Mohammadashahi et al. [64] were patients with confirmed COVID-19 stratified by sex. In that study, among patients who died, women presented with higher PLR values compared to men; however, no differences in AUC analysis were observed between the two groups (0.539 in both groups), but a difference in cut-off was found. Patients in the study groups, in addition to SARS-CoV-2 infection, also had comorbidities, which may have influenced the results.

### 4.2. Variability in Disease Severity Definitions Across Studies

In addition to the lack of standardization in study populations, the variability in AUC values may also stem from differences in the definition of disease severity. Singh et al. [53] classified patients with oxygen saturation (SaO_2_) ≥ 90% as non-severe and those with SaO_2_ below 90% as severe (AUC = 0.811), which contrasts with the classification used by Witarto et al. [54], who defined non-severe cases as those with SaO_2_ ≥ 93% and severe cases as those with SaO_2_ < 93% (AUC = 0.634). In the study by Botos et al. [47], the occurrence of septic shock served as the threshold criterion for dividing patients according to disease severity, obtaining an AUC value of 0.716. Papanikolopoulou et al. [51] and Kamjai et al. [49] used oxygen support requirements as a classification parameter, yielding comparable AUC values of 0.64 and 0.638, respectively. It is worth noting that Radkah et al. [52] investigated the relationship between the PLR and thrombotic events by dividing patients into those with PTE and those without PTE. They demonstrated that the PLR significantly predicted the occurrence of PTE in hospitalized patients with COVID-19 (AUC = 0.559). These inconsistencies in defining disease severity significantly limit the comparability of results across studies and may influence the assessment of the prognostic value of the PLR in COVID-19 patients.

### 4.3. Geographical and Ethnic Variability as Sources of Heterogeneity

Most of the studies included in our analysis originated from China (*n* = 5) [50,55,56,57,58], Indonesia (*n* = 3) [54,61,63] and Iran (*n* = 3) [52,59,64]. Therefore, race and geographical location may also represent important factors. They can influence immunological mechanisms, which in turn affect differences in disease severity and related mortality. This is reflected in the variation in AUC values observed among the analyzed studies.

### 4.4. Clinical Interpretation and Limitations of the PLR as a Dynamic Biomarker

Definitions of disease severity vary across studies, and differences in SARS-CoV-2 variants, epidemic phases, vaccination coverage, and geographical and healthcare system factors further affect patient selection and outcomes. Heterogeneity in study populations represents an important source of variability and may partly explain discrepancies in reported AUC values across studies. Total platelet counts and lymphocyte counts may be lower in elderly patients due to age-related hematological changes. Therefore, the age distribution across individual studies should be carefully considered when interpreting PLR values. Moreover, the included studies did not exclusively involve patients without comorbidities, which further limits comparability. As a result, the potential impact of underlying diseases on PLR values should also be acknowledged. Elevated PLR values have been reported in a range of chronic inflammatory and systemic conditions, including malignancies, cardiovascular diseases, viral infections, autoimmune disorders, and other pulmonary diseases. Therefore, the lack of studies focusing on patients without comorbidities represents a significant limitation in the interpretation of the PLR in COVID-19. In addition, the absence of detailed clinical background information further constrains interpretation, as vaccination status, treatment regimens, and the specific SARS-CoV-2 variant responsible for infection were not consistently reported across studies. These factors may have substantially influenced both disease severity and inflammatory marker profiles.

These sources of variability limit the comparability of AUC values and represent key limitations of our analysis, so conclusions about the predictive performance of the PLR should be interpreted with caution. An additional limitation is that our scoping review was based on the analysis of only one database (PubMed). Furthermore, no formal assessment of methodological quality or risk of bias of the included studies was performed; therefore, the findings should be interpreted as preliminary and descriptive, providing a basis for future investigations into the utility of the PLR.

Given the alterations in both immunological and hemostatic processes, which underlie immuno-thrombosis during COVID-19-Associated Coagulopathy, further research is warranted to clarify the diagnostic significance of the PLR in COVID-19 patients.

## 5. Conclusions

The PLR can serve as an indirect, composite and non-specific marker of COVID-19 progression, as it increases with disease severity. Despite differences in AUC values and cut-off points at admission, the association between the PLR and both disease severity and mortality in COVID-19 cannot be ignored. The consistent tendency for the PLR to increase with worsening disease provides an important rationale for further investigation. It may be a warning signal and assist in clinical decision-making regarding hospital admission and the need for intensive medical care. This parameter is derived from a simple complete blood count and does not require specialized equipment, making it both practical and accessible in routine clinical settings. It has been associated with worse clinical outcomes; however, its usefulness as an independent and generalizable marker remains uncertain. Additional research is needed to establish specific cut-off values and to interpret the PLR across diverse populations with COVID-19, and it is also worth noting how the PLR changes over time.

The PLR can be considered an indirect biomarker reflecting the interplay between inflammation and coagulation during COVID-19 progression. While it is not a direct marker of clinical deterioration, it indirectly reflects the involvement of both platelets and lymphocytes in immune-thrombotic processes, although its clinical utility still requires further investigation.

## Figures and Tables

**Table 1:** Table of search terms.

Database	Search Strategy	Number of Articles
PubMed	(platelet-to-lymphocyte-ratio[Title/Abstract]) AND (COVID-19[Title/Abstract])	74
PubMed	(platelet-to-lymphocyte-ratio[Title/Abstract]) AND (SARS-CoV-2[Title/Abstract])	22
PubMed	((platelet-to-lymphocyte-ratio[Title/Abstract]) AND (COVID-19[Title/Abstract])) AND (severity[Title/Abstract])	37
PubMed	((platelet-to-lymphocyte ratio[Title/Abstract]) AND (COVID-19 [Title/Abstract])) AND (mortality[Title/Abstract])	30

**Table 2:** Characteristics of included studies assessing severity in patients with COVID-19.

Author (Year)	Country	PLR Values		AUC (95% CI)	Sensitivity (%)	Specificity (%)	Cut-Off	*p*-Value
		Non-Severe Group	Severe Group					
Asan et al. (2021) [46]	Turkey	*n* = 668 PLR = 129 (70)	*n* = 27; PLR = 180 (156)	0.746 (0.629–0.862)	-	-	-	-
Botoș et al. (2022) [47]	Romania	*n* = 4; PLR = 272.98 (154.47–375.55)	*n* = 45; PLR = 229.52 (153.46–323.40)	0.716	-	-	428.49	<0.001
Cocoș et al. (2022) [48]	Romania	*n* = 183	*n* = 71	0.68 (0.58–0.77)	-	-	-	<0.0001
Kamjai et al. (2022) [49]	Thailand	*n* = 243; PLR = 158.6 (28.4–680.0)	*n* = 23; noninvasive oxygen support PLR = 194.2 (27.6–780.7); high-flow nasal cannula PLR = 250.0 (42.3–931.0); mechanical ventilator PLR = 239.6 (68.7–888.2)	0.638 (0.575–0.702)	-	-	-	-
Lin et al. (2021) [50]	China	*n* = 22; PLR = 152.2 (115.8–233.9)	*n* = 46; PLR = 284.0 (195.0–441.7)	0.787	-	-	-	-
Papanikolopoulou et al. (2025) [51]	Greece	*n* = 349 PLR = 172.6 (114.6–250.4)	*n* = 44; PLR = 268.4 (149.4–369.6)	0.64 (0.54–0.74)	59.1	76.9	262.2	0.003
Radkhah et al. (2023) [52]	Iran	*n* = 250; PLR = 205.50 [183.00–222.00]	*n* = 186; PLR = 235.50 [198.00–295.11]	0.559	81.46	29.94	120.5	0.001
Singh et al. (2023) [53]	India	*n* = 442; PLR = 133.7579	*n* = 169; PLR = 268.2517	0.811 (0.745–0.877)	70	75	189.2	0.000
Witarto et al. (2025) [54]	Indonesia	*n* = 39; PLR = 175.35 (122.36–240.06)	*n* = 39; PLR = 223.85 (161.35–382.73)	0.634 (0.463–0.805)	63.6	65.0	190.549	0.137
Wu et al. (2024) [55]	China	*n* = 673	*n* = 367	0.67 (0.64–0.70)	68	60	231	<0.001
Xia et al. (2022) [56]	China	*n* = 7; PLR = 167.31 (125.09–200.52)	*n* = 48; PLR = 316.00 (238.43–454.73)	0.797	79.12	81.82	230.44	<0.001
Xing et al. (2020) [57]	China	*n* = 53; PLR = 68.52 (124.00–203.81)	*n* = 8; PLR = 302.92 (156.21–425.08)	0.755 (0.524–0.985)	71.4	84.6	241.91	0.034
Zhang et al. (2024) [58]	China	*n* = 144; PLR = 226.99 (121.99–267.31)	*n* = 220; PLR = 425.88 (201.20–504.77)	0.714	68.9	71.1	235.489	0.001

Abbreviations: AUC (95%CI), area under the curve (95% confidence interval); PLR, platelet-to-lymphocyte ratio.

**Table 3:** Characteristics of included studies assessing mortality in patients with COVID-19.

Author (Year)	Country	PLR Values		AUC (95% CI)	Sensitivity (%)	Specificity (%)	Cut-Off	*p*-Value
		Survivors Group	Non-Survivors Group					
Abrishami et al. (2021) [59]	Iran	*n* = 83; PLR = 160.8 (124.2–219.4)	*n* = 17; PLR = 202.0 (120.7–201.2)	0.599 (0.368–0.731)	-	-	-	0.455
Acar et al. (2021) [60]	Turkey	*n* = 129; PLR = 261.5 (46.6–1628.0)	*n* = 19; PLR = 427.9 (106.0–1184.0)	0.733 (0.628–0.838)	57.8	68.9	289.90	0.01
Anwari et al. (2022) [61]	Indonesia	*n* = 54; PLR = 160.76 ± 94.797	*n* = 16; PLR = 223.55 ± 96.64	0.719	81.3	63	-	-
Botoș et al. (2022) [47]	Romania	*n* = 48	*n* = 42	0.758	-	-	428.49	0.001
Cocoş et al. (2022) [48]	Romania	*n* = 184	*n* = 70	0.69 (0.61–0.78)	-	-	-	<0.0001
Fois et al. (2020) [62]	Italy	*n* = 90; PLR = 214 (145–339)	*n* = 29; PLR = 265 (144–428)	0.572 (0.478–0.663)	59	58	240	0.26
Haryati et al. (2023) [63]	Indonesia	*n* = 292 ≥295 PLR = 67 (55.8%) <295 PLR = 225 (69.2%)	*n* = 153 ≥295 PLR = 53 (44.2%) <295 PLR = 100 (30.8%).	0.552 (0.494–0.611)	35.9	77	295	0.069
Mohammadshahi et al. (2024) [64]	Iran	male *n* = 934; PLR = 361.17 ± 800.43 female *n* = 707; PLR = 399.62 ± 559.63	male *n* = 211; PLR = 333.75 ± 319.15 female *n* = 155; PLR = 483.35 ± 748.27	0.539 (male) (0.510–0.568) 0.539 (female) (0.505–0.573)	49 (male) 54 (female)	63 (male) 60 (female)	255 (male) 247 (female)	0.079 (male) 0.151 (female)
Ortega-Rojas et al. (2022) [65]	Peru	*n* = 82; PLR = 28.2 (16.5–47.2)	*n* = 180; PLR = 43.7 (25.5–67.3)	0.697 (0.583 0.754)	62.8	69.6	34.2	0.5
Papanikolopoulou et al. (2025) [51]	Greece	*n* = 363; PLR = 172.7 (114.1–261.9)	*n* = 30; PLR = 270 (156–367.3)	0.65 (0.53–0.76)	69	68.1	229	0.009
Singh et al. (2023) [53]	India	*n* = 530; PLR = 157.6247	*n* = 81; PLR = 258.2	0.741 (0.651–0.831)	60	72.5	210.6	0.000
Witarto et al. (2025) [54]	Indonesia	*n* = 53; PLR = 192.34 (137.15–308.63)	*n* = 25; PLR = 194.74 (149.45–371.20)	0.474 (0.298–0.651)	86.7	29.6	137.674	0.783
Wu et al. (2024) [55]	China	*n* = 883; PLR = 175 (114–272)	*n* = 157; PLR = 285 (150–431)	0.66 (0.61–0.70)	74	55	268	<0.001
Zhang et al. (2024) [58]	China	*n* = 206; PLR = 284.69 (136.36–334.25)	*n* = 158; PLR = 429.08 (181.63–513.54)	0.633	62.7	63.1	254.715	0.001

Abbreviations: AUC (95%CI), area under the curve (95% confidence interval); PLR, platelet-to-lymphocyte ratio.

## Data Availability

Data sharing is not applicable to this article as no new data were created or analyzed in this study.
